# The value of luteinizing hormone basal values and sex hormone-binding globulin for early diagnosis of rapidly progressive central precocious puberty

**DOI:** 10.3389/fendo.2023.1273170

**Published:** 2024-01-22

**Authors:** Meiyu Zhang, Jun Sun, Ying Wang, Yanhui Wu, Xiaona Li, Rong Li, Yafei Fang, Hua Bai, Peiliang Luo, Yingdi Yuan

**Affiliations:** ^1^ Pediatric Endocrinology Department, The First People’s Hospital of Lianyuangang, Lianyungang Clinical College of Nanjing Medical University, Lianyungang, China; ^2^ Pediatric Endocrinology Department, Xuzhou Medical University Affiliated Hospital of Lianyungang, Lianyungang, China; ^3^ Pediatric Endocrinology Department, Postgraduate Training Base of Lianyungang First People's Hospital of Jinzhou Medical University, Liaoning, China

**Keywords:** central precocious puberty, luteinizing hormone (LH), sex hormone-binding globulin (SHBG), ROC curves, rapidly progressive central precocious puberty

## Abstract

**Objective:**

This study aimed to investigate the diagnostic value of luteinizing hormone (LH) basal values and sex hormone-binding globulin (SHBG) for rapidly progressive central precocious puberty (RP-CPP).

**Methods:**

A total of 121 girls presenting with secondary sexual characteristics were selected from the Department of Pediatric Endocrinology, Lianyungang Clinical Medical College of Nanjing Medical University, from May 2021 to June 2023. The children were followed up for 6 months and were divided into three groups: RP-CPP group (n=40), slowly progressive central precocious puberty (SP-CPP) group (n=40), and premature thelarche (PT) group (n=41). The differences in LH basal values and SHBG among girls in the three groups were compared. ROC curves were drawn to analyze the value of LH basal values and SHBG in identifying RP-CPP.

**Results:**

Significant differences were observed in age, height, predicted adult height (PAH), weight, body mass index (BMI), bone age (BA), BA-chronological age (CA), LH basal, LH peak, FSH basal, LH peak/FSH peak, estradiol (E2), testosterone, and SHBG levels between the RP-CPP group and the SP-CPP and PT groups (P < 0.05). The LH basal value in the RP-CPP group was higher than that in the SP-CPP group and the PT group, while SHBG levels were lower than in the latter two groups, and these differences were statistically significant (P < 0.05). When the LH basal value was ≥0.58 IU/L and SHBG was ≤58.79 nmol/L, the sensitivity for diagnosing RP-CPP was 77.5% and 67.5%, and the specificity was 66.7% and 74.1%.

**Conclusion:**

Detection of basal LH and SHBG levels allows for early diagnosis of the progression of central precocious puberty.

## 1 Introduction

Central precocious puberty(CPP) is a common endocrine disorder in children. CPP has a significant impact on an individual’s health and development. It can result in accelerated skeletal maturation, advanced bone age, and premature epiphyseal closure, which can ultimately influence adult height. Moreover, CPP is associated with an increased susceptibility to conditions such as obesity, type 2 diabetes, breast cancer, and cardiovascular diseases (1). CPP can be classified into two types: slowly progressive CPP (SP-CPP) and rapidly progressive CPP (RP-CPP). SP-CPP is defined as partial early development of secondary sexual characteristics before the age of puberty, with possible acceleration of sexual developmental progression, linear growth, and bone age greater than chronological age. However, the bone age/height age ratio does not increase in this group of children and is not damaging in predicting adult height. Children with this type require close clinical follow-up and prompt intervention if abnormalities are detected. On the other hand, RP-CPP is distinguished by a heightened rate of growth and rapid bone development, and accelerated development of secondary sexual characteristics. These children may experience a significant advancement of bone age compared to their chronological age in a short period. This leads to early menarche and impaired adult height. In such cases, the treatment involves administering a gonadotropin-releasing hormone agonist (GnRHa) (2). Currently, the luteinizing hormone releasing hormone (LHRH) stimulation test stands as a crucial diagnostic tool for CPP (3). However, determining whether some patients require GnRHa treatment still requires long-term follow-up observation, involving repeated blood tests to measure hormone levels. The administration of the LHRH stimulation test is known for its time-consuming nature and high cost, and patients’ compliance with it may be poor. To find a more convenient and less invasive test, this study aims to investigate the diagnostic significance of baseline luteinizing hormone (LH) and sex hormone-binding globulin (SHBG) in early RP-CPP.

## 2 Materials and methods

### 2.1 Clinical subject study

A cohort of 121 girls, who displayed early onset of secondary sexual characteristics and sought consultation at the Pediatric Endocrinology Department at the Lianyungang Clinical Medical College, Nanjing Medical University, between May 2021 and June 2023, were included in this study. Inclusion criteria are as follows: (1) age ≤ 8 years; (2) The onset of puberty without menarche; (3) completion of the GnRH stimulation test; (4) no prior treatment with any medication for precocious puberty. Exclusion criteria are as follows: (1) peripheral precocious puberty or secondary precocious puberty caused by organic lesions; (2) obesity or low birth weight; (3) concomitant liver, kidney, thyroid, adrenal insufficiency, or other systemic diseases; (4) a history of using medications that could potentially influence the hypothalamic-pituitary-gonadal axis before their visit.

### 2.2 Grouping

In order to avoid missed diagnosis of children who did not seem to have a rapid progressing puberty at the time of initial diagnosis, these children were followed up for a period of 6 months. The children underwent physical, radiologic, and biochemical examinations at the time of initial visit and at 6 months. Based on the clinical data at the time of initial consultation and during the 6-month follow-up period, the 121 study participants were divided into three groups: the Rapidly Progressive Central Precocious Puberty (RP-CPP) group (n=40) (nine of these children did not show rapidly progressive precocious puberty at the time of first diagnosis, but progressed within 6 months), the Slowly Progressive Central Precocious Puberty (SP-CPP) group (n=40), and the Premature Thelarche (PT) group (n=41). The criteria for inclusion in the CPP groups were as follows (4): (1) girls developed secondary sexual characteristics before 8 years old; (2) pelvic ultrasound showed uterus and ovaries enlarge, several follicles ≥4 mm of diameter in the ovaries; (3) GnRH stimulation test revealed that LH>5 IU/L, LH peak /FSH peak ratio >0.6; (4) bone age (BA) was more than one year ahead of chronological age; (5) the presence of linear growth acceleration and more rapid annual growth rates than in healthy children of the same age. Girls meeting one or more of the following conditions were included in the RP-CPP group, otherwise they were included in the SP-CPP group (5): (1) accelerated sexual development, Tanner stage of breast development progressed to next stage within 6 months; (2) rapid progression of BA, indicated by BA/chronological age (CA) ratio > 1; (3) significantly increased growth rate, height growth of more than 4cm in 6 months. The inclusion criteria for the Premature Thelarche group were: (1) breast development without any other signs of sexual development; (2) no significant acceleration in growth or advancement of skeletal development; (3) results of the GnRH stimulation test: LH peak < 5U/L, LH peak/FSH peak ratio < 0.6.

### 2.3 Clinical data and laboratory examination

The clinical data analyzed for the study participants at their initial visit included age of onset, height, weight, target height(TH), body mass index (BMI), predicted adult height (PAH), BA, BA-CA (BA minus CA). We used a fully automated chemiluminescent immunoassay analyser from Beckman for the detection of baseline LH levels, LH peak levels, baseline follicle-stimulating hormone (FSH) levels, FSH peak levels, testosterone, estradiol (E2), and SHBG. The minimally detectable concentrations of LH, FSH, E2, testosterone and SHBG were classified as 0.2 IU/L, 0.8 IU/L, 15 pg/ml, 10 ng/dl and 11.44 nmol/L.

### 2.4 Statistical analysis

The collected data were subjected to statistical analysis using SPSS version 27.0 software. For normally distributed quantitative data, the results are expressed as mean ± standard deviation (x ± SD), and intergroup comparisons were conducted using one-way analysis of variance (ANOVA). Non-normally distributed data are presented as median (interquartile range) [M (P25, P75)], and intergroup comparisons were performed using the Friedman test.Categorical data between different groups were compared using the chi-square test. To evaluate the diagnostic potential of baseline LH and SHBG in diagnosing RP-CPP, ROC curve analysis was employed, and the optimal diagnostic threshold was determined based on the cutoff value corresponding to the maximum Youden’s index. A p-value of less than 0.05 was considered statistically significant for all analyses.

## 3 Results

### 3.1 Analysis of clinical data

Significant statistical differences (P < 0.05) were observed among the RP-CPP group, SP-CPP group, and PT group in various parameters, including age, height, predicted adult height (PAH), weight, BMI, BA, BA-CA, baseline LH levels, peak LH levels, baseline FSH levels, LH peak/FSH peak ratio, E2, testosterone, and SHBG levels. After conducting pairwise comparisons, it was observed that the RP-CPP group exhibited significantly older age, greater height, PAH, weight, BMI, BA, BA-CA, and testosterone levels compared to both the SP-CPP group and PT group (P < 0.05). Additionally, the RP-CPP group had lower SHBG levels than the SP-CPP group and PT group, also with statistically significant differences (P < 0.05). However, no statistically significant differences (P > 0.05) were found between the SP-CPP group and PT group in any of these parameters.Furthermore, in terms of E2 levels, both the RP-CPP group and SP-CPP group showed higher levels than the PT group, and this difference was statistically significant (P < 0.05). However, there were no statistically significant differences (P > 0.05) in E2 levels between the RP-CPP group and SP-CPP group. Regarding baseline FSH levels, the RP-CPP group had significantly higher levels than the PT group (P < 0.05), but there were no significant differences (P > 0.05) between the RP-CPP group and SP-CPP group, nor between the SP-CPP group and PT group.Furthermore, the RP-CPP group and SP-CPP group exhibited higher baseline LH levels, peak LH levels, and LH peak/FSH peak ratio than the PT group, and the RP-CPP group had higher levels than the SP-CPP group (P < 0.05). However, there were no statistically significant differences (P > 0.05) among the three groups concerning genetic height and peak FSH levels (**Table 1**).

**Table 1:** Comparison of clinical data at the time of initial diagnosis in three groups of children.

Variable	RP-CPP	SP-CPP	PT
Age[M(P_25_, P_75_), years]	8.0 (7.5,8.0)^ab^	7.0 (6.5,7.5)	7.0 (6.0,7.0)
Height[(x̅ ± s), cm]	135.8 ± 6.4^ab^	128.4 ± 6.5	125.9 ± 7.4
TH[(x̅ ± s), cm]	159.8 ± 3.9	161.7 ± 3.2	161.2 ± 3.8
PAH[(x̅ ± s), cm]	158.2 ± 6.3^ab^	164.7 ± 8.8	163.7 ± 7.6
Weight[M(P_25_, P_75_), kg]	33.0(29.0,38.2)^ab^	26.3(23.2,30.2)	24.0(22.05,27.4)
BMI[M(P_25_, P_75_), kg/m^2^]	17.6(16.3,19.8)^ab^	15.9(14.9,17.2)	15.3(14.4,16.8)
BA[M(P_25_, P_75_), years]	11.0(10.0,11.0)^ab^	9.0 (7.0,9.0)	8.0 (7.0,9.0)
BA-CA[M(P_25_, P_75_), years]	3.0 (2.0,3.0)^ab^	1.5 (0.1,2.0)	1.5 (0.3,2.0)
Baseline LH value [M(P_25_, P_75_),IU/L]	1.22(0.60,2.45)^ab^	0.69(0.25,1.13)^a^	0.20(0.20,0.50)
LH peak value [M(P_25_, P_75_), IU/L]	17.26(11.24,25.52)^ab^	9.31(6.52,11.89)^a^	3.22(2.85.3.97)
Baseline FSH value [M(P_25_, P_75_), IU/L]	4.21(2.67,6.10)^a^	3.63(2.54,5.70)	3.00(2.35,4.32)
FSH peak value [M(P_25_, P_75_), IU/L]	17.72(13.28,20.35)	17.50(15.03,21.55)	18.3(15.8,23.16)
LH peak value**/**FSH peak value [M(P_25_, P_75_)]	0.99(0.63,1.53)^ab^	0.53(0.37,0.71)^a^	0.18(0.14,0.23)
E2[M(P_25_, P_75_),pg/ml]	20.56(15.00,40.36)^a^	16.35(15.00,24.74)^a^	15.00(15.00,15.00)
testosterone [M(P_25_, P_75_),ng/dl]	18.50(11.19,24,72)^ab^	10.00(10.00,14.24)	10.00(10.00,12.79)
SHBG[M(P_25_, P_75_),nmol/L]	52.15(42.63,79.38)^ab^	70.15(51.33,98.23)	72.90(59.95,94.35)

TH for target height, PAH for predicted adult height, BMI for body mass index, BA for bone age, BA-CA for bone age minus chronological age, LH for luteinizing hormone, FSH for follicle-stimulating hormone, E2 for estradiol, SHBG for sex hormone-binding globulin.In the context of "a," the values were found to be statistically significant (P < 0.05) when compared with the PT group. Similarly, in relation to "b," the values also showed statistical significance (P < 0.05) when compared with the SP-CPP group.

### 3.2 Correlation analysis of various parameters among the three groups

Pearson correlation analysis revealed that baseline LH value was positively correlated with BMI, LH peak value, LH peak/FSH peak value among the three groups of patients (P < 0.01). Moreover, baseline LH and BMI were significantly negatively correlated with SHBG (P < 0.01). Please refer to **Tables 2**, **3** for details.

**Table 2:** Correlation analysis between baseline LH value and BMI, LH peak value, LH Peak/FSH Peak value and SHBG in the three groups of patients.

Variable	r value	P value
BMI	0.319	<0.01
LH peak value	0.602	<0.01
LH peak value**/**FSH peak value	0.616	<0.01
SHBG	-0.272	<0.01

**Table 3:** Correlation analysis of SHBG with BMI and baseline LH value in the three groups of patients.

Variable	r value	P value
BMI	-0.484	<0.01
Baseline LH value	-0.272	<0.01

### 3.3 Analysis of the diagnostic efficacy of baseline LH and SHBG levels in diagnosing RP-CPP

**Figure 1:**
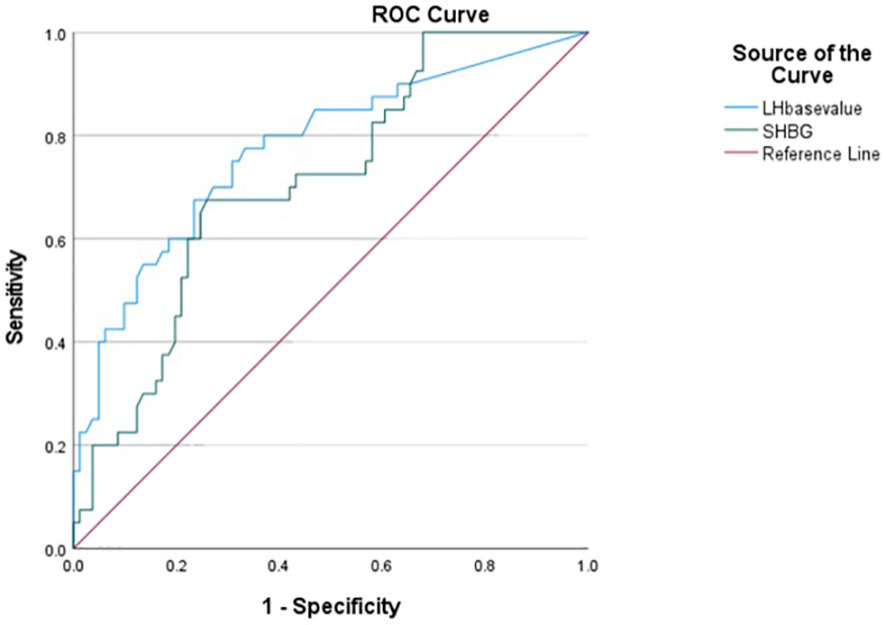
ROC curves for prediction of baseline LH and SHBG levels for RP-CPP (the optimal thresholds for baseline LH and SHBG were 0.58 IU/L and 58.79 nmol/L, which gave sensitivities for diagnosing RP-CPP was 77.5% and 67.5%, and the specificity was 66.7% and 74.1%).

The ROC analysis results for diagnosing RP-CPP based on baseline LH and SHBG values were obtained, and the area under the curve (AUC), sensitivity, and specificity were calculated. The optimal cutoff values for baseline LH and SHBG were found to be 0.58 IU/L and 58.79 nmol/L, respectively, where the corresponding Youden’s index was maximized. At these cutoff values, the sensitivity for diagnosing RP-CPP was 77.5% and 67.5%, and the specificity was 66.7% and 74.1%, respectively. Please refer to **Table 4**; **Figure 1** for detailed results.

**Table 4:** Efficacy analysis of baseline LH values and SHBG levels for diagnosis of RP-CPP.

Variable	Cut point	AUC	95%CI	Sensitivity(%)	Specificity(%)	Youden’s J index
LH base value	0.58IU/L	0.773	0.681~0.866	77.5%	66.7%	0.442
SHBG	58.79nmmol/L	0.711	0.617~0.805	67.5%	74.1%	0.416

[AUC] area under the curve; [95% CI] 95% confidence interval.

## 4 Discussion

CPP stands as a prevalent endocrine disorder in children, and its pathogenesis involves the premature activation of the hypothalamic-pituitary-gonadal (HPG) axis, resulting in augmented secretion of luteinizing hormone and follicle-stimulating hormone, thereby causing the development of secondary sexual characteristics (4). If CPP is not promptly diagnosed and treated, it may not only impact the final adult height but also lead to related psychological issues.The primary objective of this study was to identify a swift and convenient diagnostic approach, thus establishing a solid theoretical foundation for clinical diagnosis and treatment. Ultimately, the goal is to enhance the management of precocious puberty in affected children, streamlining their care and support. LH is a member of the glycoprotein hormone family, synthesized and secreted by the anterior pituitary gland. It is jointly regulated by GnRH (gonadotropin-releasing hormone) and ovarian hormones, exhibiting pulsatile secretion. LH, along with FSH, co-regulates the synthesis and secretion of estrogen, progesterone, and other hormones, participates in maintaining the normal menstrual cycle (6). Elevated LH is one of the important indicators of HPGA (hypothalamic-pituitary-gonadal axis) activation. In recent studies, a baseline LH greater than 0.2 IU/L was proposed as an indicator of the initiation of puberty (7). In a study by Cao et al. (8),during the investigation of the diagnostic potential of basal luteinizing hormone in central precocious puberty, it was noted that a baseline LH value greater than 0.535 mIU/L could serve as a reliable marker for CPP diagnosis, with a sensitivity of 68.9% and specificity of 86.0%, obviating the need for a GnRH stimulation test to confirm CPP. Results from Vurallı et al. (9) revealed that during early breast development, a baseline luteinizing hormone level of ≥0.65 IU/L serves as a sensitive indicator of activation in the hypothalamic-pituitary-gonadal axis and could aid in the early detection and diagnosis of related hormonal changes. Lee et al. (10) demonstrated that due to the diurnal rhythm of early LH secretion during puberty, the morning unstimulated LH (mLH) is more sensitive in predicting CPP. When mLH ≥ 0.22 IU/L, the sensitivity for CPP screening was 69.4%, and the specificity was 82.1%. Calcaterra et al. (2) determined that a baseline LH value of ≥0.2 IU/L exhibited a significant association with the onset of RP-CPP, and highlighted the potential of using LH levels as a diagnostic marker to identify cases of RP-CPP early on. Wankanit et al. (11)showed that LH basal values greater than or equal to 0.2 IU/L provided a preliminary determination of whether puberty had been initiated in Tanner II and Tanner III girls. The different studies they reviewed had various cut-off values due to some factors, including different study populations, methods of measuring LH, and time of obtaining samples. Therefore it is hardly possible to establish a specific threshold value that is universally adaptable. The baseline LH values they proposed are more applicable to their own study groups. In this study, a comparison was made between the results of the GnRH stimulation test for the three groups of patients. The findings indicated that the RP-CPP group had significantly higher baseline LH levels compared to the SP-CPP group and PT group. Notably, when the baseline LH level reached ≥ 0.58 IU/L, the corresponding Youden’s index was maximized, demonstrating a sensitivity of 77.5% and specificity of 66.7%. These results underscore the potential of using the baseline LH level as an effective marker for differentiating RP-CPP from other conditions.

SHBG is a homodimeric peptide chain composed of amino acid residues, capable of regulating the metabolic clearance of hormones such as 17β-estradiol and testosterone (12). SHBG possesses a single binding site and selectively binds and transports sex hormones. By regulating the concentration of free hormones, SHBG alters the interaction between sex hormones and their receptors. Additionally, SHBG concentrations are modulated by various conditions, including puberty, polycystic ovary syndrome (PCOS), insulin resistance, and disorders of glucose and lipid metabolism (13–15). Relatively high concentrations of SHBG bind low sex hormone levels in young, prepubertal children. As adolescents enter puberty, sex hormone levels increase while plasma SHBG levels decline (16). The results of this study show that the E2 and testosterone levels in the RP-CPP group are higher than those in the PT group, while SHBG levels are lower than those in the PT group. However, there was a difference in age between the three groups of girls, the girls in the RP-CPP group being 1-2 years older than the other two groups. A report stated that SHBG levels change with age, with a variation of about 6-7 nmol/L in SHBG levels in healthy children aged 6-8 years (17). With pubertal development, the SHBG gradually decreases. However, we found that the decline in SHBG levels between the RP-CPP versus the other 2 groups was greater than expected for the age difference, i.e. the older age of the RP-CPP group. Early puberty development is marked by a simultaneous rise in body fat, and obesity can promote leptin secretion, which acts on the HPG axis, increases LH pulse rate and levels, and accelerates the initiation of puberty. Furthermore, SHBG is negatively correlated with body fat mass and insulin levels in adolescents during puberty (18–20). Obesity is strongly associated with early puberty in girls. Changes in body composition occur very early, and fat mass has already increased in most cases by the time of diagnosis. Obese children often experience insulin resistance, leading to reduced glucose utilization in tissues and organs. SHBG is regulated by insulin, and insulin resistance decreases its synthesis and secretion. Sørensen et al. (17)showed lower levels of SHBG in girls with idiopathic CPP compared to age-, body mass index-, and puberty-matched controls. In this study, by comparing the clinical data of the three groups of girls, it was observed that the BMI of RP-CPP girls was significantly higher than that of the SP-CPP group and PT group, while SHBG levels were lower than that of the two groups. Pearson correlation analysis showed a negative correlation between BMI and SHBG, suggesting a close relationship between the increase in BMI and the decrease in SHBG levels in RP-CPP girls, which is concomitant with the rapid progression of puberty. However, there is limited literature on whether SHBG can be used as a diagnostic indicator for RP-CPP. This study calculated ROC curves and found that when SHBG levels were ≤58.79 nmol/L, the sensitivity and specificity for diagnosing RP-CPP were 67.5% and 74.1%, respectively, indicating that SHBG has some predictive value for the progression of central precocious puberty.

The diagnostic process for RP-CPP is complex and diverse, and is not based on a single laboratory test. Because of the different testing methods, and other variables (such as time of the day when LH is measured), LH and SHBG values obtained at the time of the initial testing may not be entirely reproducible on subsequent testing. Therefore, the diagnosis of RP-CPP should be based on the child’s medical history, physical examination and ancillary tests.

In conclusion, baseline LH and SHBG can serve as relevant indicators for determining rapidly progressive central precocious puberty. When the baseline LH is ≥0.58 IU/L and SHBG is ≤58.79 nmol/L have satisfactory predictive value for rapidly progressive central precocious puberty, and can provide useful guidance for planning of more complex diagnostic testing and eventual treatment.

## Data availability statement

The original contributions presented in the study are included in the article/supplementary material. Further inquiries can be directed to the corresponding authors.

## Ethics statement

The studies involving humans were approved by Ethics Committee of the First People’s Hospital of Lianyungang. The studies were conducted in accordance with the local legislation and institutional requirements. The participants provided their written informed consent to participate in this study.

## Author contributions

MZ: Writing – original draft, Data curation. JS: Project administration, Writing – original draft. YW: Data curation, Writing – original draft. YHW: Data curation, Investigation, Writing – review & editing. PL: Methodology, Supervision, Validation, Writing – review & editing. YY: Methodology, Project administration, Supervision, Validation, Writing – review & editing. XL: Data curation, Investigation, Writing – original draft. RL: Data curation, Investigation, Writing – original draft. YF: Investigation, Writing – review & editing. HB: Investigation, Resources, Writing – review & editing.
